# Dental Miscellanies

**Published:** 1872-11

**Authors:** H. Scott


### "Dental Miscellanies."

BY H. SCOTT.

Editor Register: Your contributor, W. E. Driscoll, in the October Register, seems excited to the point of fierceness over some suggestions of mine on oxychloride of zinc as a therapeutic agent. His gentle urbanity towards myself needs no reply. Something, however, is due to the readers of your monthly. Time will vindicate me.

The writer puts forward, as an apology for his interference, that those who have previously taken the trouble to answer so well my views may tire of the "*endless task*," and for a time allow me to claim a triumph. To this it is only necessary to answer, that it is wisdom to retire from the work of a bootless task. There is a class of men dispersed through society, who, in every sentence they speak or write, labor to keep the *Ego* always in the foreground, and whose constant effort is to establish the impression that what they do not know is not known at all. This kind of egotism is found in all ranks of life. I once asked a watch maker if his time-piece was right. The question came near being the end of him. The presumption that could ask a time-keeper if his time was right, was beyond all bounds.

My critic thinks the "*boy*" who lost his incisor that had been filled with gold, without the intervention of a non-conducting substance, should claim damages. I will give the gentleman one more case, so that, when he comes up to award damages, both the injured parties may come in on equal footing.

A young lady of about twenty years had a central incisor filled about three years since, under circumstances very much like those of case No. 1, referred to, with the single exception that the case was more favorable, as I now recall it. In this case I used the oxychloride carefully and well. The case did well for more than six months, and I was pleased with the encouragement it afforded. At length she came and said the tooth ached a little. A week later she called again, with a frightfully swelled face, and was at the time suffering greatly. I extracted the tooth; and after cracking it open found the nerve cavity filled with effete grumous fluid of most offensive fætor.

The dental student of "*one year's experience*," who has gained more truth than my thirty-five years have given me, in regard to the reproduction of tooth substance, is very felicitous in his attainments; still I am too obtuse to go backward, or to see my way out of the fog of the author's and my own inadequate experience, and must reaffirm that, dentition once finished, there is no further tooth growth, W. E. Driscoll to the contrary notwithstanding. I do not accept the deposit of solid matter in the nerve cavity upon the recession of the nerve before the advance of attrition of the teeth (which after all I believe I have noticed in a few cases), as evidence of the reproduction of dentine. Has the gentleman placed this subtance under his microscope and found it organic, as dentine proper. But this deposit takes place in some cases only, while in others the tooth wears away, until the bleeding nerve is brought to the dentist for relief. At best it is but a last feeble rally of the *vis medicatrix naturæ*, as is sometimes manifested in dying vegetables. But when the gentleman charges me with saying that this deposit never takes place, he perpetrates either a very ignorant or a very willful misstatement. I have said that the teeth are never reproduced in whole, or in part, by a purely physiological function, and I say so now. And if solid arches have ever been thrown over exposed nerves in contact with oxychloride, the same substance has been deposited in the nerve cavities a thousand times to one in the absence of any foreign substance, as in the instance of the wearing down of the teeth already referred to.

I do not doubt that my theories are "*utterly perverse and absurd*" to the learned gentleman; but I think they will cease to be so after a while. And I wish I could devise some way to quiet his fears about the harm likely to be done by my teaching, which he thinks may deter others from learning to save nerves alive. At present I can do no more, from my unenlightened stand-point, than to suggest that the future may show that he may have misled as many as myself, by his over-weaning confidence in a practice now no more than on trial. But finally, at the close of our friend's little effort, he snaps the cord at one full swoop, by saying, because he could say no better, that the "*errors*" of these "dental miscellanies" are too numerous, and impliedly too absurd, to be noticed in detail, and intimates that they ought to be written down; well, then, he should write them down.

It must nevertheless be a matter of regret to all dentists who feel any interest in the question, that the gentleman has neither given nor attempted to give any proof of his satisfaction with the use of the os-artificial. He says he has no proof to give—has never seen a case where the nerve was bridged over where it had been used. But he does say that his cases are all doing so well he can't afford to pull the teeth out and split them open to please me. Well, that is a real poser—a perfect silencer. But if he has one hundred cases where he has applied oxychloride in direct contact with the nerve membrane, of one year's standing or more, I would like, were it possible, to have the entire hundred laid open. I would like to guarantee Dr. Driscoll that not five living nerves could be found in the hundred.

But, Mr. Editor, I respectfully refer readers to my remarks in the August REGISTER, and ask them if there is one word written there that merits the tirade of your belligerent contributor. I know there is a class of persons, somewhat numerous, who already know so much that to attempt to know more would be folly, and to question whose *finalities* would be irreverent and unpardonable presumption. I feel that I should not be marshaled with them, for I am yet a student who is willing to learn. I have lived through an era of experiments in dental science, and have tried them all, but found few finalities. If men have reached finalities, why are they forever asking questions? We do not need to ask questions of others if we know enough ourselves. What does friend Driscoll mean when he uses the words, "*deterred from learning to save pulps?*" If it is known how to save them, that is enough. It is exactly, I opine, because we don't know how, that we are still discussing the oxychloride. None would rejoice more than myself over its triumph. It is because I have not found it the *sine qua non* in the case of exposed tooth nerves, that I have said one word about it. And I hope that even Mr. Driscoll himself will be gracious enough to allow me to say what my experiences have been, as well as to theorize a little, provided there be no danger of "*deterring*" some one from adopting *his* views.

I have said that I use the oxychloride. I do use it. I use it to cap nerves, and use it to quiet sensitive dentine; but I am using it as ever body else is, without *knowing* what is to be its position after awhile. I am waiting. I can afford to wait. All are waiting, and watching, and hoping. But while we would all like to save nerves alive, we must accept facts. We will not attempt any of the fixed laws of either organic or inorganic matter; we will not presume to try to cause inorganic matter to become organic and vital. And this brings us back to the oxychloride on its own account.

I have previously said that this agent seemed to be a devitalizer. I still believe so. It is in every way, relatively to living tissue, a foreign substance. It is more; it is an irritant. If not, why do dentists find it necessary to interpose collodion, and the like. One of my former critics believed its successes and failures referable to the skill or otherwise of the hands that applied it. I asked him in return whether, in his opinion, the action of arsenic on the mucus membrane would be modified by the skill, or otherwise, of the hands that caused it to pass into the human stomach, but heard no more from him. I have before said that, in some cases, this agent caused painless death of the nerve. Of that I am still satisfied. In such cases the devitalized nerve, with its membranes, may be carried away by the absorbents; or, on the other hand, decomposed, forming a putrescent mass which, for the want of outlet, would not fail to induce periodontitis, with more or less disastrous consequences; and all this in accordance with the degree of health and strength of the individual, or the degree and force of the *vis medicatrix naturæ.*

Therapeutically there is nothing either rational or scientific in its use, but everything to the contrary; and if the nerve lives, and the bone grows in juxta-position with it, I premise that the instances will be rare and far between. But I never have said, and do not now say to any body, don't use the oxychloride. We will use it just as the medical profession has in time past used hundreds of supposed remedies, because they did not know of anything better, but which are forever now laid aside. We shall all have seen, ten years from now, how many of the teeth with capped nerves will have festered and been removed from the mouth, or on the other hand, remain in their sockets performing the work assigned them in the economy of life. If successful, all right; if a failure, we shall have learned one more lesson.

I do not deny that I felt stung by the uncivil and ungentlemanly attack of Dr. Driscoll in the October number. He is a stranger to me, and I to him. His language transcends the lines of either professional or civil intercourse, and I repel it as such. I write no opinions I do not feel fully competent to stand by. Time will set all things right. Meanwhile I shall always be among the first to acknowledge an error when proven to be such.

And now, having said so much, I say that nothing can induce me to return again to the oxychloride but another unjust assault. But nothing shall hinder me from the freest and fullest expression of my experiences and opinions, whenever it is my pleasure to do so. With all courtesy I am ready to learn or unlearn.

